# Role of astroglial Connexin 43 in pneumolysin cytotoxicity and during pneumococcal meningitis

**DOI:** 10.1371/journal.ppat.1009152

**Published:** 2020-12-28

**Authors:** Chakir Bello, Yasmine Smail, Vincent Sainte-Rose, Isabelle Podglajen, Alice Gilbert, Vanessa Moreira, Fabrice Chrétien, Martine Cohen Salmon, Guy Tran Van Nhieu

**Affiliations:** 1 Team Intercellular Communication and Microbial Infections, Center for Interdisciplinary Research in Biology, Collège de France, Paris, France; 2 Institut National de la Santé et de la Recherche Médicale, Paris, France; 3 Centre National de la Recherche Scientifique, Paris, France; 4 MEMOLIFE Laboratory of excellence and Paris Science Lettre, Paris, France; 5 Team Physiology and physiopathology of the gliovascular unit, Center for Interdisciplinary Research in Biology, Collège de France, Paris, France; 6 Experimental Neuropathology Unit, Institut Pasteur, Paris, France; University of Birmingham, UNITED KINGDOM

## Abstract

*Streptococcus pneumoniae* or pneumococcus (PN) is a major causative agent of bacterial meningitis with high mortality in young infants and elderly people worldwide. The mechanism underlying PN crossing of the blood brain barrier (BBB) and specifically, the role of non-endothelial cells of the neurovascular unit that control the BBB function, remains poorly understood. Here, we show that the astroglial connexin 43 (aCx43), a major gap junctional component expressed in astrocytes, plays a predominant role during PN meningitis. Following intravenous PN challenge, mice deficient for aCx43 developed milder symptoms and showed severely reduced bacterial counts in the brain. Immunofluorescence analysis of brain slices indicated that PN induces the aCx43–dependent destruction of the network of glial fibrillary acid protein (GFAP), an intermediate filament protein specifically expressed in astrocytes and up-regulated in response to brain injury. PN also induced nuclear shrinkage in astrocytes associated with the loss of BBB integrity, bacterial translocation across endothelial vessels and replication in the brain cortex. We found that aCx4-dependent astrocyte damages could be recapitulated using *in vitro* cultured cells upon challenge with wild-type PN but not with a *ply* mutant deficient for the pore-forming toxin pneumolysin (Ply). Consistently, we showed that purified Ply requires Cx43 to promote host cell plasma membrane permeabilization in a process involving the Cx43-dependent release of extracellular ATP and prolonged increase of cytosolic Ca^2+^ in host cells. These results point to a critical role for astrocytes during PN meningitis and suggest that the cytolytic activity of the major virulence factor Ply at concentrations relevant to bacterial infection requires co-opting of connexin plasma membrane channels.

## Author summary

The role of non-endothelial cells constituting the neurovascular unit during infectious meningitis is poorly appreciated despite their key regulatory functions on the blood-brain barrier integrity. Here, we show that *Streptococcus pneumoniae* or pneumococcus, a major causative agent of bacterial meningitis, targets astroglial cells to translocate across brain endothelial vessels. We found that astroglial connexin 43, a gap junctional component, played a major role during PN meningitis in mice. PN translocation and replication in the brain cortex were associated with connexin-dependent fragmentation of astrocytic the GFAP network, a process associated with brain injury. These findings were recapitulated and extended *in vitro* using cultured primary astrocytes and the major PN virulence determinant Pneumolysin. Ply-mediated cytotoxicity was linked to Ca^2+^ increase and required aCx43, arguing against a direct toxin activity. The results reveal a key role for astroglial signaling during PN crossing of the BBB and shed light on the mechanism of Ply-mediated cytotoxicity during meningitis.

## Introduction

PN is a bacterial commensal of the human nasopharynx and pathogen responsible for invasive diseases, including septicaemia and meningitis with high morbidity worldwide [1,2]. The large majority of *in vitro* studies on PN translocation across the BBB have been performed using cultured brain endothelial cells that do not reflect the complexity of the BBB. Brain endothelial cells form the mechanical and highly selective barrier isolating the brain cortex from the bloodstream [3]. The BBB selectivity is permitted by the endothelial expression of restrictive and specific transport systems, a poor pinocytic activity, and tightly sealed intercellular junctions [3,4]. Other cell types forming the neurovascular unit regulate the BBB function [4,5]. Among these, astrocytes, the most abundant cells of the Central Nervous System (CNS), form glial heterogeneous networks in various brain compartments that regulate neural and vascular functions, in particular BBB maturation and maintenance [6–8]. Astrocytes may regulate the BBB function through signaling controlling the tightness endothelial cell junctions [9]. Also, astrocytes form the *glia limitans*, a physical barrier limiting exchanges between the brain parenchyma and the subarachnoid space as well as between the parenchyma and parenchymal vessels [3]. This latter barrier, also referred to as *glia limitans perivascularis*, consists of astrocyte processes called end-feet that entirely cover the basal membrane ensheathing the vessels of the brain parenchymal vasculature [9,10]. At this interface, astrocytes modulate the integrity and functions of the BBB, neuroinflammation, cerebral blood flow and interstitial fluid drainage [10–12].

Pertinent to these functions, CNS astrocytes are highly interconnected via Connexin (Cx) channels [13]. The Cx family consists of 21 structurally and functionally conserved proteins reported to date in mammals, designated by their apparent molecular weight [14]. CNS astrocytes predominantly express Cx43 and Cx30, and to a minor extent Cx26 [13]. At gap intercellular communicating junctions, Connexins assemble into hexamers that appose to other hexamers of adjacent cells to form constitutively open channels allowing the intercellular exchange of ions and metabolites, critical for the K^+^ and glutamate neuro-modulatory buffering functions of astrocytes [13]. Connexin hexamers also form constitutively closed hemichannels at the plasma membrane that can open upon stimulations such as increase of intracellular Ca^2+^, oxidative or mechanical stress and inflammation [13]. Hemichannels have been shown to release ATP, glutamate, and nicotinamide adenine nucleotide (NAD) that act in a paracrine manner. In pathological conditions, massive ATP release by hemichannels and overload of intracellular Ca^2+^ lead to cytotoxicity of astrocytes and other neighboring cells [15].

Despite current evidence for the role of astrocytes in the regulating the BBB, their role during its crossing by bacterial meningitis-causing microorganisms has been poorly investigated. By directly acting as a physical barrier or by indirectly controlling endothelial cell junctions, perivascular astrocytes may prevent bacterial translocation into the cortex and severe brain injury associated with encephalitis. Also, the glia limitans is believed to form a continuous structure isolating the brain parenchyma from the subarachnoid space and brain parenchymal blood capillaries [6]. Thus, through the release of mediators in the cerebrospinal fluid (CSF) or perivascular spaces regulating endothelial cell junctions, astrocytes may regulate bacterial translocation events across meningeal or parenchymal blood vessels. During mice meningitis, PN was found to adhere to subarachnoid vessels during the first hours of infection, prior to its progressive association with brain parenchymal vessels during the 14H post-infection period [16]. However, in these studies, PN counts in the subarachnoid space did not increase concomitantly during the infection period, questioning whether bacterial translocation across blood vessels preferentially occurred at these specific brain areas.

Numerous PN meningitis-associated factors have been reported that target host cell receptors, degrade the extracellular matrix or intercellular junctional components, trigger inflammatory responses and allow escape from innate defense mechanisms [1,2,17]. PN may cross the BBB via a paracellular route, following the loosening of endothelial cell junctions triggered by Ply and inflammatory responses [17]. PN can also transcytose through brain vascular endothelial cells by targeting the Platelet-Activating Factor receptor (PAFR) and the laminin receptor [18]. Other receptors such as the Platelet Endothelial Cell Adhesion Molecule (PECAM-1) and the poly-Immunoglobulin Receptor (pIgR) could be involved [2,19,20], the latter being also targeted by PN during crossing of the nasopharyngeal epithelium [21]. Interactions between PN and cell receptors require down-regulation of the bacterial capsule, essential for bacterial survival in the blood and evasion of phagocytosis [2]

Ply is a critical virulence factor involved in PN invasive diseases. Through various actions including cytotoxicity linked to its pore-forming toxin activity, complement activation, and binding to Toll-like Receptors, Ply was reported to promote tissue inflammation and damages, as well as high bacteremial titers [22,23]. The role of Ply in PN crossing of the BBB is a mater of debate. Ply may favor PN translocation by triggering inflammation [24,25] known to promote BBB permeabilization [4]. However, there are conflicting reports about the role of Ply in promoting brain inflammation [2,22]. Depending on studies, Ply may or may not be required for bacterial replication and meningeal inflammation [26–29] By interfering with complement activity, Ply is required to resist bacterial clearance to promote high bacteremial titers, therefore rendering complex the determination of its precise role during meningitis [30–32]. Arguing for a direct role of Ply in brain inflammation and BBB permeabilization, intravenous administration of Ply at concentrations similar to those encountered during PN meningitis leads to BBB permeabilization [25,33]. Of note, high levels of Ply expression may antagonize with PN transcytosis accross the BBB by, and meningitis-causing PN strains express lower Ply levels than those causing sepsis [34]. Ply was shown to alter astrocyte shape, glutamate signaling and to trigger synaptic damage at non-lytic concentrations and during PN meningitis [35–37]. However, the role of astrocytes during PN translocation across the BBB remains ill defined.

## Results

### PN co-opts aCx43 during meningitis

**Fig 1:**
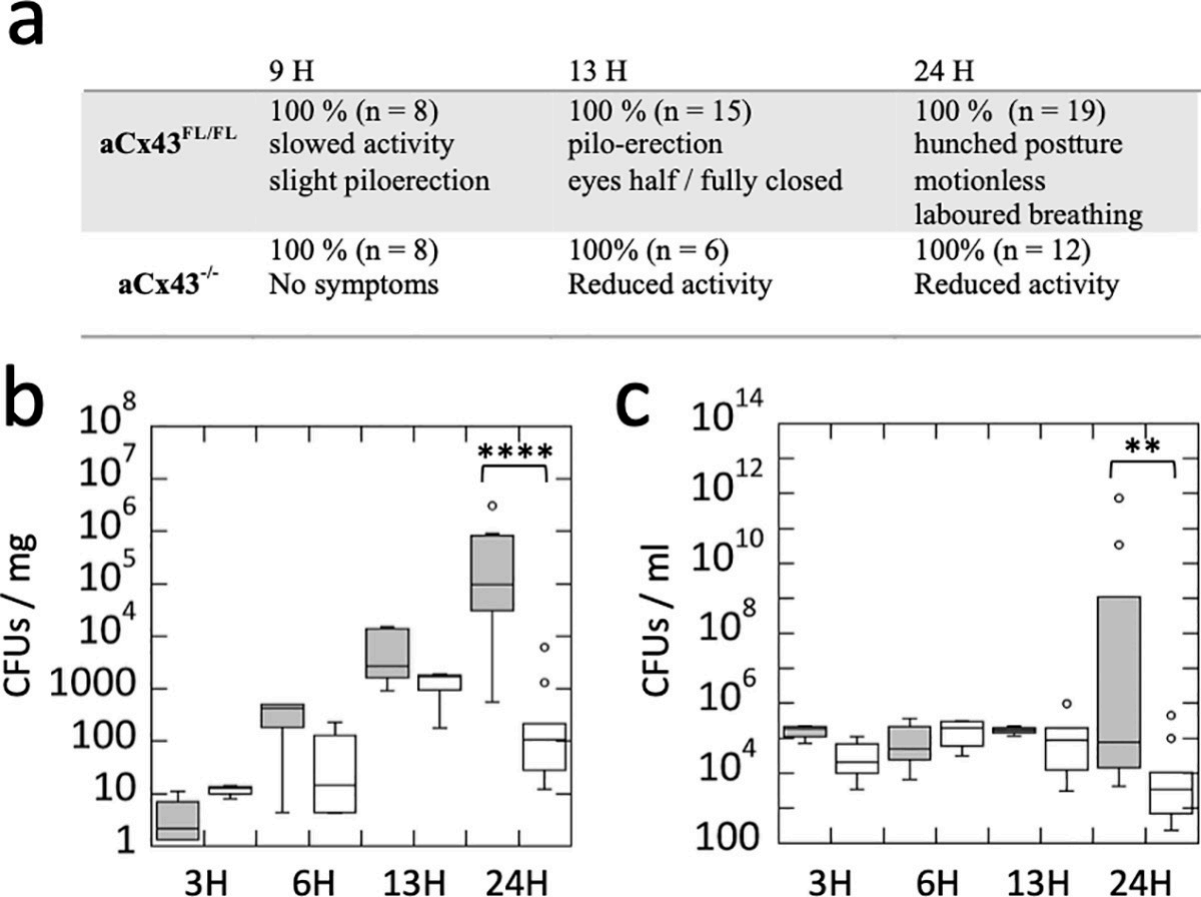
Astroglial Cx43 aggravates PN meningitis. **a**, symptoms of C57/BL6 mice proficient (aCx43^FL/FL^) or deficient (aCX43^-/-^) for astroglial Cx43 following intravenous PN challenge. **b**, **c**, box plots showing medians of CFU determination associated with brain (B) or blood (C) samples at the indicated post-infection time. Grey boxes: aCx43^FL/FL^ mice; empty boxes: aCX43^-/-^mice. **b**, aCx43^FL/FL^: 3H, N = 4; 6H, N = 4, 13H, N = 5; 24H, N = 12. aCx43^-/^: 3H, N = 4; 6H, N = 4, 13H, N = 3; 24H, N = 12. **c**, aCx43^FL/FL^: 3H, N = 4; 6H, N = 4, 13H, N = 5; 24H, N = 9. aCx43^-/-^ 3H, N = 4; 6H, N = 4, 13H, N = 3; 24H, N = 9. Wilcoxon test. **: p = 0.01; ****: p < 0.0001.

Using mouse retro-orbital vein injection, we found that all aCx43^FL/FL^ control mice developed meningitis when challenged with TIGR4, a PN serotype 4 wild-type strain [38]. All PN infected mice showed reduced activity, associated with piloerection at 9 H post-infection, with aggravating symptoms including hunched postures and absence of motility at 24 H post-infection, the longest incubation time at which mice were sacrificed to limit animal suffering (Figs 1A and S1). As shown in Fig 1B, CFU determination in brains of aCx43^FL/FL^ mice indicated that bacterial translocation was detected as early as 3 H post-injection and increased exponentially to reach a median value of 6.2 x 10^4^ CFUs / mg after 24 H, a time point at which rupture of the BBB integrity could be detected macroscopically (S1A Fig). Bacterial CFUs could be detected in the cerebrospinal fluid at 24H but not at 3H post-infection, suggesting that PN did not translocate preferentially across meningeal vessels at early infection stages (S1B Fig). In control experiments, aCx43^FL/FL^ mice showed no symptoms when infected with a control non-pathogenic *Escherichia coli* K12 strain and no CFU counts could be detected in brains sampled at 24 H post-infection (N = 4). Interestingly, aCx43^-/-^ mice did not present obvious symptoms besides a slightly reduced activity up to 24 H post infection, suggesting that infection was controlled. Accordingly, PN translocation in the brain of aCx43^-/-^ mice was significantly decreased with a median value of 1.2 x 10^2^ CFUs / mg at 24 H post-infection. aCx43^FL/FL^ and aCx43^-/-^ mice shared similar high bacteremial median values between during the first 13H incubation. However, consistent with severe sepsis, at 24 H post-infection, some aCx43^FL/FL^ mice showed PN blood titers that were up to 4 logs higher than titers in aCx43^-/-^ mice (Fig 1C). PN infection led to up-regulation of the pro-inflammatory cytokines TNF-α and IL1-β, as well as of endothelial inflammation markers as revealed by qRT-PCR, but to a similar extent in aCx43^FL/FL^ and aCx43^-/-^ mice despite the difference in bacterial titers (S1C Fig).

### aCx43-dependent killing of astrocytes and loss of BBB integrity during PN translocation across the endothelial vessels

**Fig 2:**
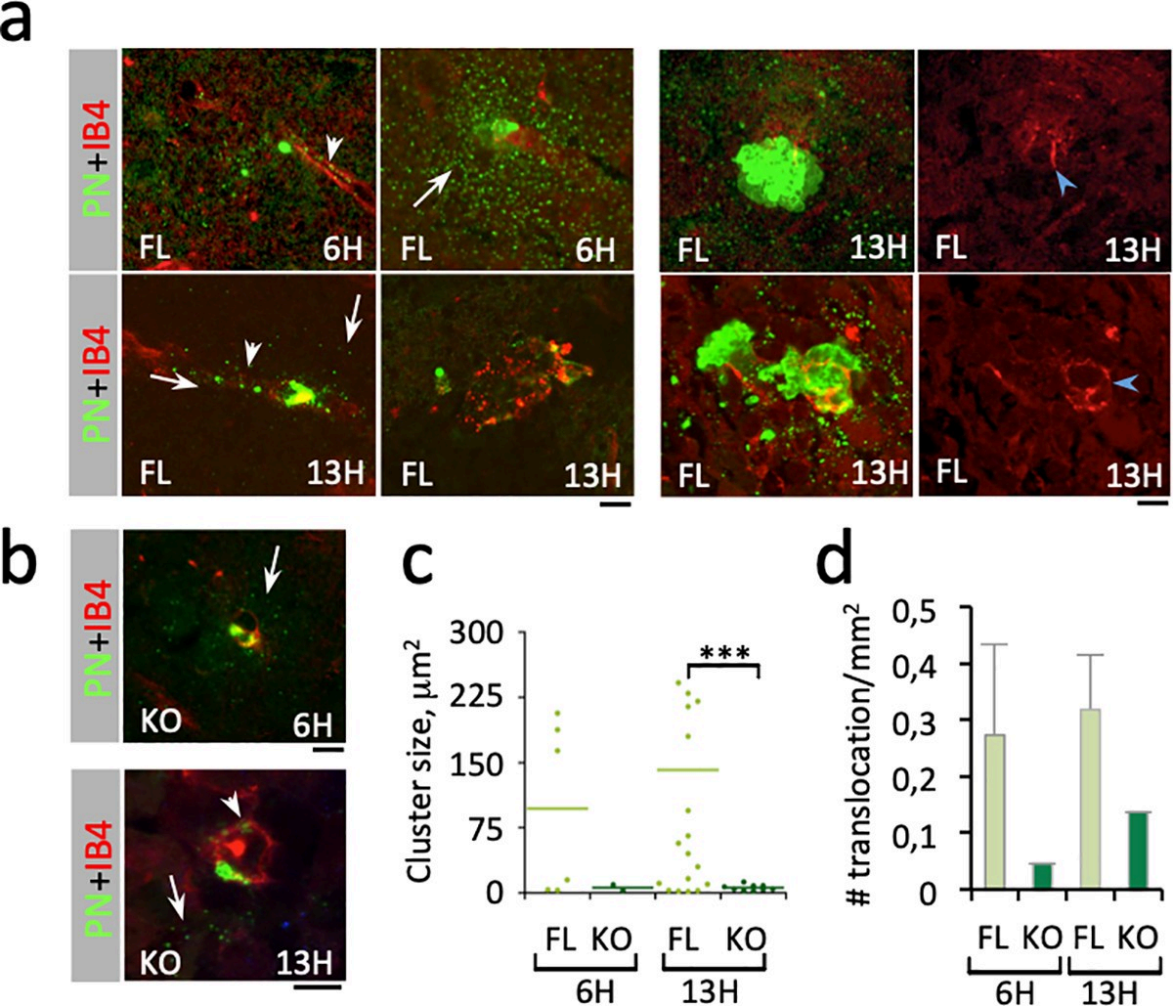
PN translocation across the BBB is increased by aCx43 expression. Immunofluorescence analysis of brain slices from PN-infected mice. The time post-infection is indicated. Scale bar = 5 μm. **a**, **b**, representative micrographs. Red: IB4-labeling of endothelial vessels; green: PN capsule. FL: aCx43^FL/FL^ mice; KO: aCx43^-/-^ mice. Arrows: capsular remnants in the brain cortex. Arrowheads: capsular remnants in vessels. **a**, right panels: blue arrowheads point at vessel damages. **c**, size of translocated PN microcolony. 6H: FL, N = 3, 6 foci; KO, N = 3, 2 foci. 13H: FL, N = 3, 20 foci; KO, N = 3, 9 foci). **d**, frequency of bacterial translocation events per mm^2^ of brain slice (N = 2, n = 900 60x microscopy fields). **c, d,** median values are indicated.

To further characterize the early events associated with PN translocation across the BBB, we performed brain immunofluorescence analysis (Materials and Methods). No bacteria were detected in the brain cortex at 3 H post-infection and only 0.7 to 3.3 bacteria or bacterial clusters / mm^2^ were observed at 6 H and 13 H post-infection. We did not detect preferential PN translocation in a specific brain region. At 6H post-infection, bacteria were detected in the subarachnoid space, as well as cortical and deeper parenchymal area, but not in the choroid plexus where bacteria were detected only after 13H post-infection. As shown in Fig 2A, however, bacteria were always found in close association with brain vessels, consistent with early brain translocation events. Immuno-labeling revealed the presence of small capsular remnants at the vicinity of bacteria, including in brain vessels and cortex, consistent with bacterial lysis or capsular shedding reported during *in vitro* interaction with endothelial cells also occurring *in vivo* (Fig 2A, arrowhead; [17,39]). Remarkably, such capsular remnants were also detected in the brain cortex seemingly leaking from vessels associated with translocated bacteria at 6H post-infection, consistent with loss of vessel integrity at early stages of crossing of the BBB by PN (Fig 2A, arrows). Capsular remnants and loss of endothelial vessel integrity was clearly detected in association with bacterial clusters at 13H post-infection. Consistent with the scoring of CFUs from sampled brains, aCx43^-/-^ mice showed three times less translocation events compared to aCx43^FL/FL^ mice as early as 6H post-infection (Fig 2B and 2C). Single or a discrete number of bacteria were observed in aCx43^-/-^ mice, with lesser capsular remnants and vessel destruction, consistent with poor PN translocation across the BBB (Fig 2D). Bacterial cluster size quantification also indicated that PN brain intra-cortical replication was higher in aCx43^FL/FL^ relative to aCx43^-/-^ mice, with clusters 6 and 23-times bigger at 6H and 13 H post-infection, respectively (Fig 2C and 2D).

**Fig 3:**
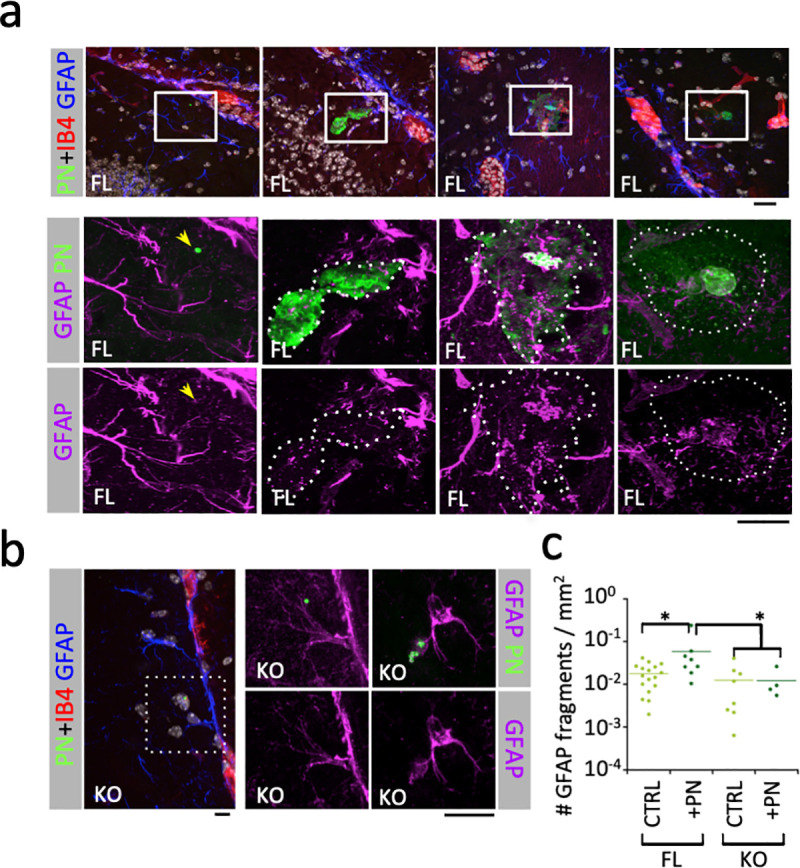
PN induces the aCx43-dependent destruction of astrocytic GFAP network. Immunofluorescence analysis of brain slices from PN-infected mice. The time post-infection is indicated. Scale bar = 5 μm. **a, b**, representative micrographs. Red: IB4-labeling of endothelial vessels; green: PN capsule; purple: GFAP. Higher magnifications of the insets in the top panels (**a**) or left panel (**b**) are shown. **a**, FL: aCx43^FL/FL^ mice. Arrow: GFAP association with a single translocated bacterium. **b**, KO: aCx43^-/-^ mice. **c**, quantification of GFAP fragments per μm^2^ in area corresponding to capsular shedding (dotted area) associated with translocated bacterial microcolony. FL CTRL, N = 2, > 10 000 fragments; FL+PN, N = 2, 1658 fragments; KO CTRL, N = 2, 3619 fragments; KO+PN, N = 2, 263 fragments. Mann and Whitney. *: p < 0.05.

To analyze the effects of PN infection on CNS astrocytes, brain slices were immuno-labeled for the prototypical marker glial fibrillary acid protein (GFAP), the major intermediate filament protein in astrocytes. GFAP is a marker of astrocytes’ activation in response to CNS injury [40]. GFAP also controls the astrocyte shape and is implicated in various functions, including their neuro-modulatory and BBB regulatory functions [41,42]. Strikingly, PN translocation and intracortical growth in aCx43^FL/FL^ mice brains were associated with major astrocyte injury characterized by the destruction of the cytoskeletal GFAP network. GFAP debris occurred in close contact with intracortical PN microcolonies and at the vicinity defined by the area corresponding to shed capsular materials (SCMs) (Figs 3 and S2, dotted area; S1 Video). These observations suggested that secreted bacterial products were responsible for the cytotoxicity of astrocytes in the absence of direct bacterial contact. Furthermore, astrocytes at the vicinity of these bacterial microcolonies also showed nuclear shrinkage described for the PN pore-forming toxin Ply (S3 Fig, [43]). In contrast, astrocyte injury as quantified by the formation of GFAP debris was not observed in aCx43^-/-^ mice brains (Figs 3C and S3). Instead, rare and discrete bacteria were detected in association with intact GFAP-labeled astrocytic processes (Fig 3B).

These results indicate that PN translocation across brain vessels is associated with a local loss of BBB integrity and with aCx43-dependent astrocytic death. The occurrence of aCx43-dependent astrocyte killing at a distance from intact bacteria suggests the aCx43-dependent cytotoxic action of a secreted bacterial product during PN crossing of the BBB.

### Ply mediates aCx43-dependent killing of astrocytes

**Fig 4:**
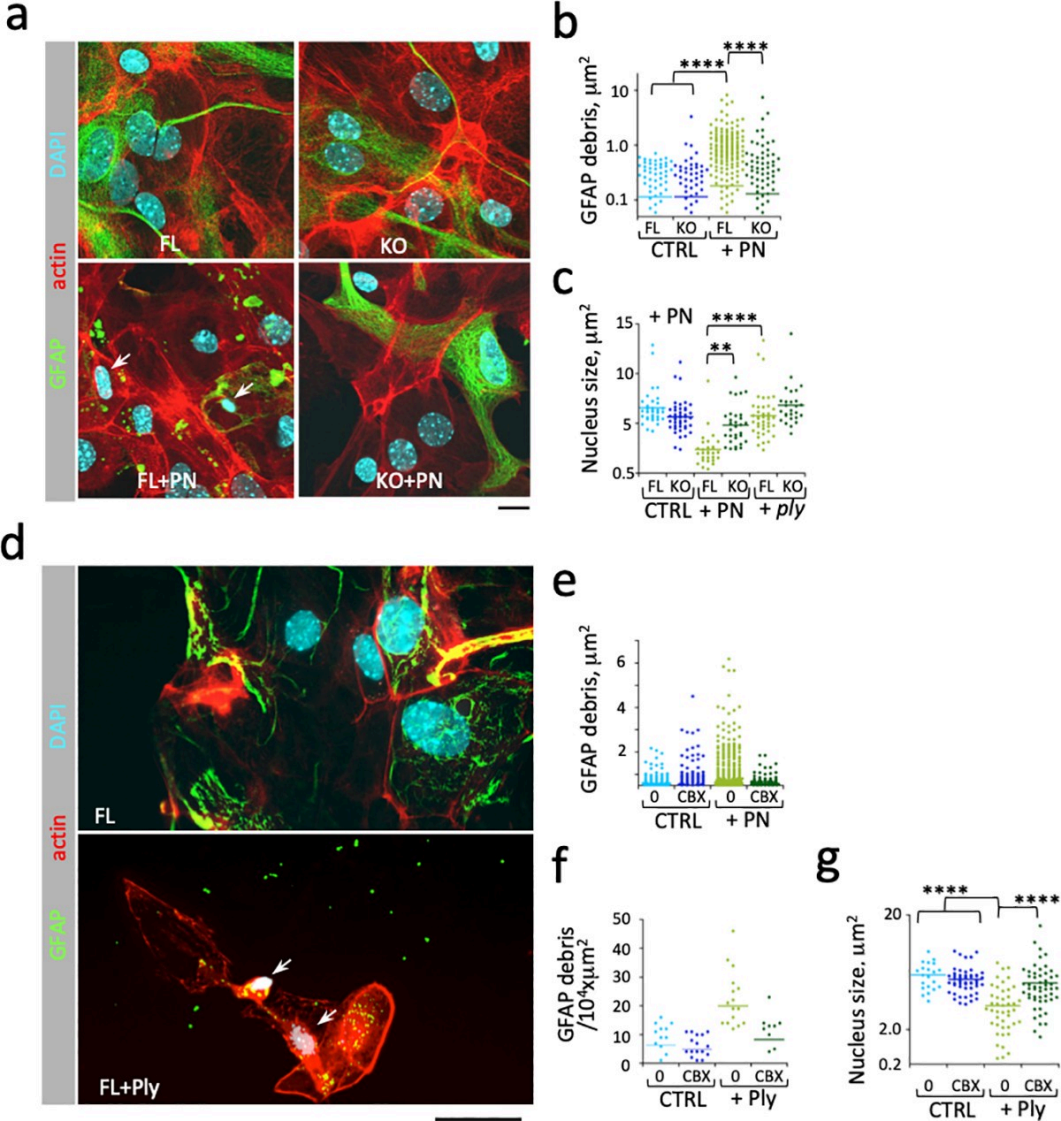
Ply mediates aCx43-dependent astrocytic death. Primary astrocytes derived from wild-type (FL) or aCx43^-/-^ (KO) mice were challenged with wild-type TIGR4 (PN) or an isogenic *ply* mutant (*ply*) (**a-c**), or purified Ply at the indicated concentrations for 60 min (**d-g**). Samples were fixed and processed for immunofluorescence staining of GFAP (green), F-actin (red) or nuclear DNA using DAPI (cyan). Cbx: challenge in the presence of 100 μM carbenoxolone. Scale bar = 5 μm. **a**, **d**, representative micrographs. Arrows point at nuclear shrinkage. **b**, **e**, quantification of GFAP debris size. **c**, **g**, nuclear size quantification. **f**, quantification of the number of GFP debris per 10^4^ x μm^2^ field. Bars: median values. N = 2, > 35 nucleus, > 1500 GFAP debris. Mann-Whitney. **: p < 0.01; ****: p < 0.0001.

Ply is a well-characterized cholesterol-dependent cytolysin (CDC) forming large pores into host cell membranes that are generally thought to account for its cytotoxicity [22]. Therefore, the involvement of aCx43 in PN- or Ply-mediated astrocyte toxicity was intriguing. To investigate this, mouse cortical primary astrocytes were isolated and challenged *in vitro* with PN (Materials and Methods). As observed in brain slices, PN challenge for 90 min led to a clear destruction of the GFAP network associated with the formation of GFAP debris in cultured aCx43^FL/FL^ astrocytes following PN-challenge, which was not detected in aCx43^-/-^ astrocytes (Fig 4A and 4B). Also consistent with in vivo results, the formation of GFAP debris was associated with nuclear shrinking and cell retraction in aCx43^FL/FL^ astrocytes (Fig 4A and 4C, FL+PN). Since these observations were reminiscent of cytotoxicity linked to a pore-forming toxin, we tested a PN isogenic mutant deficient for *ply* expression. Strikingly, nuclear shrinkage was not observed in aCx43^-/-^ astrocytes challenged with wild-type PN, nor in aCx43^FL/FL^ astrocytes challenged with the *ply* mutant, indicating a role for aCx43 in Ply-mediated cytotoxicity (Fig 4C, FL+PN). Consistent with this, nuclear shrinkage was not detected in aCx43^FL/FL^ astrocytes in the presence of the Cx channel inhibitor carbenoxolone (Fig 4G). As expected, a Ply-deficient isogenic PN mutant did not induce any detectable nuclear shrinkage in aCx43^-/-^ astrocytes (Fig 4C, KO + *ply*).

To further demonstrate the role of aCx43 in Ply-mediated cytotoxicity, we purified Ply in a recombinant form (Materials and Methods) and used it to challenge astrocytes. As shown in Fig 3E–3G, the destruction of GFAP network and nuclear shrinkage induced by PN were phenocopied when cells were challenge with purified recombinant Ply at a concentration of 250 nM. These Ply-mediated effects were inhibited by carbenoxolone (Fig 3F–3G).

### Cx43 expression confers Ply-mediated cytotoxicity linked to plasma membrane permeabilization

**Fig 5:**
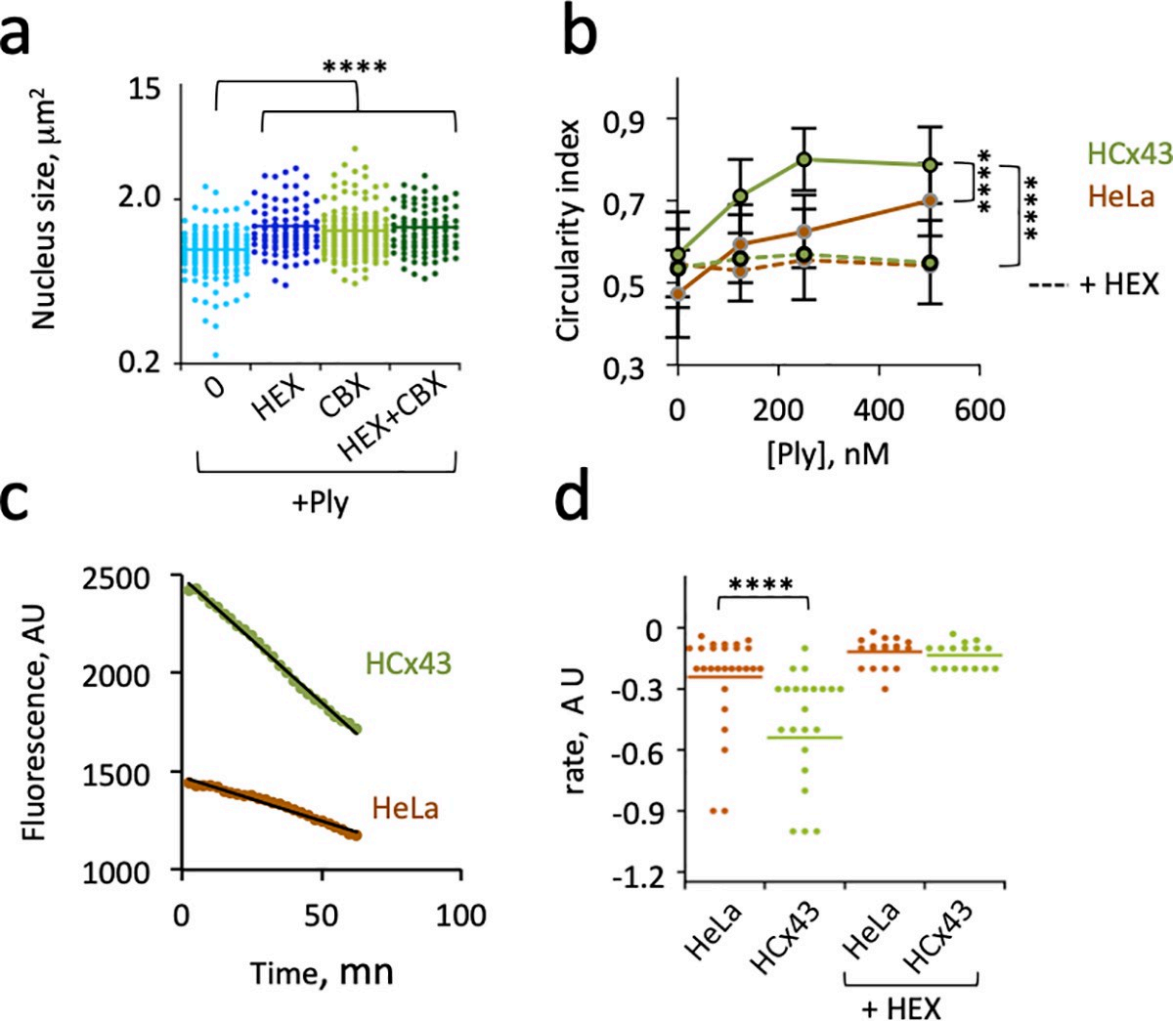
Cx43 expression confers Ply-mediated cytotoxicity. Cells were challenged for 90 min with purified Ply (+Ply) at 250 nM or at the indicated concentration. **a**, wild-type asytrocytes. **b**-**f**, HeLa cells or stable transfectants expressing Cx43 (HCx43). Incubation in the presence of: +HEX: hexokinase; + CBX: 100 μM Carbenoxolone. a, b, Cells were fixed and processed for the quantification of nuclear (**a**) or cell retraction (**b**). Median values are represented. > 25 cells per sample. Mann-Whitney. ****: p < 0.001. **d**, > 72 cells per sample. ANCOVA. ****: p < 0.001. **c**, **d**, calcein-loaded cells were incubated with 250 nM Ply. **c**, representative experiment of Ply-induced calcein release. **d**, rates of calcein decrease determined from linear fits as in “**c**” (Materials and Methods). Bars: median values. Mann-Whitney. ****: p < 0.001.

Our results suggested that Cx43 play a major role in Ply-mediated cytotoxicity. These findings are reminiscent of cytotoxicity induced by the small pore forming RTXs toxins involving the release of ATP through plasma membrane channels formed by purinergic P2X7 receptors and pannexins [44,45]. To test this, astrocytes were incubated in the presence of hexokinase to deplete ATP from the extracellular milieu [45]. As shown in Fig 5A, Ply-mediated cytotoxicity was inhibited by hexokinase treatment to an extent similar to that of carbenoxolone, suggesting a role for ATP released through Cx43 hemichannels. To further confirm the implications of Cx43 in Ply-mediated cytotoxicity and extend these findings to cells other than astrocytes, we analyzed the effects of Ply in HeLa cells that do not express known Cxs and HeLa cells stably transfected with Cx43 (HCx43) [46]. As shown in Figs 5B and S4, challenge with PN did not lead to detectable change in morphology of parental HeLa cells, but cell retraction was clearly observed for HCx43 cells. As expected, cell retraction was dependent on Ply, since it was not detected upon challenge with a *ply* mutant and was induced by purified Ply (Figs 5B and S4). As observed for astrocytes, Ply-induced rounding of HCx43 cells was inhibited by hexokinase (Fig 4B). We next performed dye release assays to test the role of ATP release through Cx43 hemichannels in Ply-mediated plasma membrane permeabilization. As shown in Fig 5C and 5D, upon challenge with Ply, the rates of calcein fluorescence decrease were significantly higher in HCx43 cells compared to parental HeLa cells indicative of higher plasma membrane permeabilization linked to Cx43 expression. Furthermore, the Cx43-dependent plasma membrane permeabilization was abrogated in the presence of hexokinase during Ply challenge, consistent with a role for ATP release through Cx43 hemichannels (Fig 5D).

### Role of Cx43 expression in Ply-mediated Ca^2+^ increase

At agonist concentrations, extracellular ATP (eATP) acts in a self-feeding amplifying loop that permeabilizes plasma membranes by stimulating Ca^2+^ responses that lead to the opening of hemichannels and further release of ATP in the extracellular medium [14,15]. Our results suggest that such a Cx43-dependent amplifying loop plays a critical role in previously described Ply-mediated cytotoxicity linked to Ca^2+^ overload [43,47].

**Fig 6:**
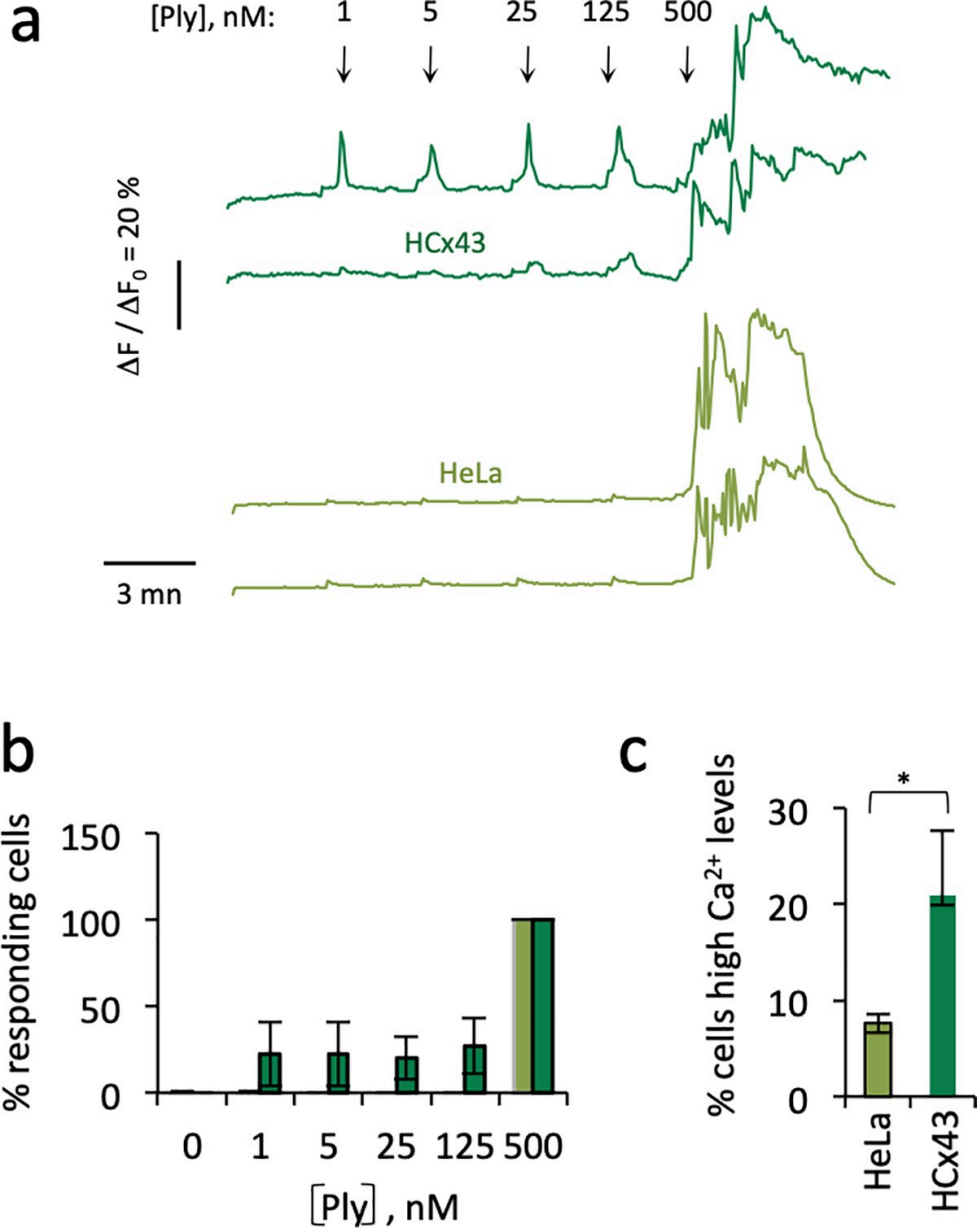
Role of Cx43 in Ply-mediated Ca^2+^ increase. Cells were loaded with the Ca^2+^ indicator Fluo-4 and Ca^2+^ imaging was performed following addition of purified Ply at the indicated concentrations. **a**, representative traces of Ca^2+^ variations in single cells. The arrows indicate the addition of Ply at the indicated concentrations. **b**, **c**, light green bars: HeLa cells; dark green bars: HCx43 cells. **b**, percent of cells showing Ca^2+^ responses. HeLa cells; N = 3, 145 cells. HCx43 cells. N = 3, 107 cells. **c**, percent of cells with lasting high Ca2+ levels following treatment with 500 nM Ply. HeLa cells, N = 3, 130 cells. HCx43 cells, N = 3, 89 cells. Kruskal-Wallis. *: p = 0.049.

To test this, we performed Ca^2+^ imaging to study the role of Cx43 at different Ply concentrations. As shown in Fig 6A and 6B, low concentrations of Ply triggered Ca^2+^ responses in HCx43 cells, with ca 25% of cells showing Ca^2+^ responses at Ply concentrations ranging from 1 to 125 nM, while no responses were observed in HeLa cells at these concentrations (Fig 6B). Furthermore, all HCx43 and HeLa cells showed responses at a Ply concentration of 500 nM, (Fig 6B), but Ply triggered a lasting increase in intracellular Ca^2+^ that did not return to basal levels consistent with Ca^2+^ overload, three-times more frequently in HCx43 cells compared to HeLa cells (Fig 6C).

Together, these results indicate that Cx43-mediated signaling amplifies Ply-mediated Ca^2+^ signals and favors sustained responses, consistent with Ca^2+^ overload and cytotoxicity linked to plasma membrane permeabilization observed in Cx43-expressing cells.

## Discussion

Our results indicate a role for aCx43 in the PN translocation across the BBB associated with a loss of brain vascular endothelial integrity and revisit the cytotoxic role of Ply at pathophysiological concentrations. aCx43, the major Cx expressed in astrocytes, forms hemichannels involved in Ca^2+^ signaling particularly relevant for the regulation of the BBB permeability [14,15]. As a Cholesterol-Dependent Cytolysin (CDC), Ply is believed to cause cytotoxicity through the formation of large pores in host cell plasma membranes [22,48]. Our findings, however, support the notion that at physiological concentrations, Ply cytotoxicity involves Cx43 hemichannels and the release of extracellular ATP to promote host cell permeabilization. Published reports show that while high concentrations, of Ply lead to plasma membrane destabilization, low concentrations induce the formation of small pores in host cell membranes, linked to the formation of incomplete rings or arcs [49,50]. Thus, our results argue that pathophysiological concentrations, Ply functions in a manner similar to small pore forming RTX toxins shown in other systems to require host cell plasma membrane channels such as Pannexins, the P2X7 purinergic receptors and ATP release to induce hemolysis [51]. Because aCx43 is a predominant regulator of astrocyte functions, the characterization of Cx43 as major actor in Ply-mediated cytotoxicity has important implications during PN meningitis [10,14].

Ply was previously shown to induce actin cytoskeletal alterations through Ca^2+^ influx and activation of the small GTPase Rac1[35]. Ply also mediates the bundling of microtubules independent of Ca^2+^ influx [52]. Here, we show that aCx43 determines the destruction of the GFAP filament network by Ply. In other studies, the destruction of GFAP filaments has been associated with activation of Ca^2+-^dependent proteases [53,54]. The increased stimulation of Ply-mediated Ca^2+^ signals may therefore account to the *in vivo* destruction of GFAP structures linked to aCx43 expression during PN translocation in the brain. Our findings that aCx43-deficient mice are virtually resistant to PN meningitis highlight the key role of astrocytes in the regulation of the BBB integrity during infection. These findings argue for a major role of aCx43-mediated signaling in severe brain injuries linked to vascular inflammation, infarction and thrombosis of brain parenchymal vessels, often associated with death or severe neurological sequelae during PN meningitis in humans [55]. The results also show the aggravating role of astrocyte and aCx43-mediated signals in the PN translocation across parenchymal blood vessels. As opposed to parenchymal vessels, subarachnoid blood vessels are not ensheathed by the *glia limitans*. The relevance of aCx43-mediated signaling for the potential translocation of PN across meningeal vessels therefore remains to be determined. Clearly, the implications of our findings in the development of PN meningitis will require further investigation. Interestingly, Ply was also reported to be required for cardiac microlesions adjacent to blood vessels induced by PN translocation across the vascular endothelium [56]. In light of our findings, it would be interesting to determine whether Ply and Cx43-mediated signaling also play a role in the PN translocation across cardiac endothelial cells and cytotoxicity towards cardiomyocytes since Cx43 is also highly expressed in these cell types [57]. Of note, Ply was reported to induce different types of cell death, depending on its concentration, cell types or signals linked to bacterial phagocytosis [23,43,58,59]. Reminiscent of our findings, Ply-mediated necroptosis in macrophage and lung epithelial cells was linked to plasma membrane permeabilization and Ca^2+^ influx [60,61]. Prolonged intracellular cytosolic Ca2+ increase in response to PFTs has been described to trigger various types of cell death including apoptosis or necrosis, depending on possible co-stimuli, cell repair mechanisms and Ca2+ kinetics [62]. Because Cx43 regulates the kinetics of Ca2+ responses induced by Ply, its expression levels and organization in hemichannels may represent an important factor determining Ply-induced cell death in different cell types.

**Fig 7:**
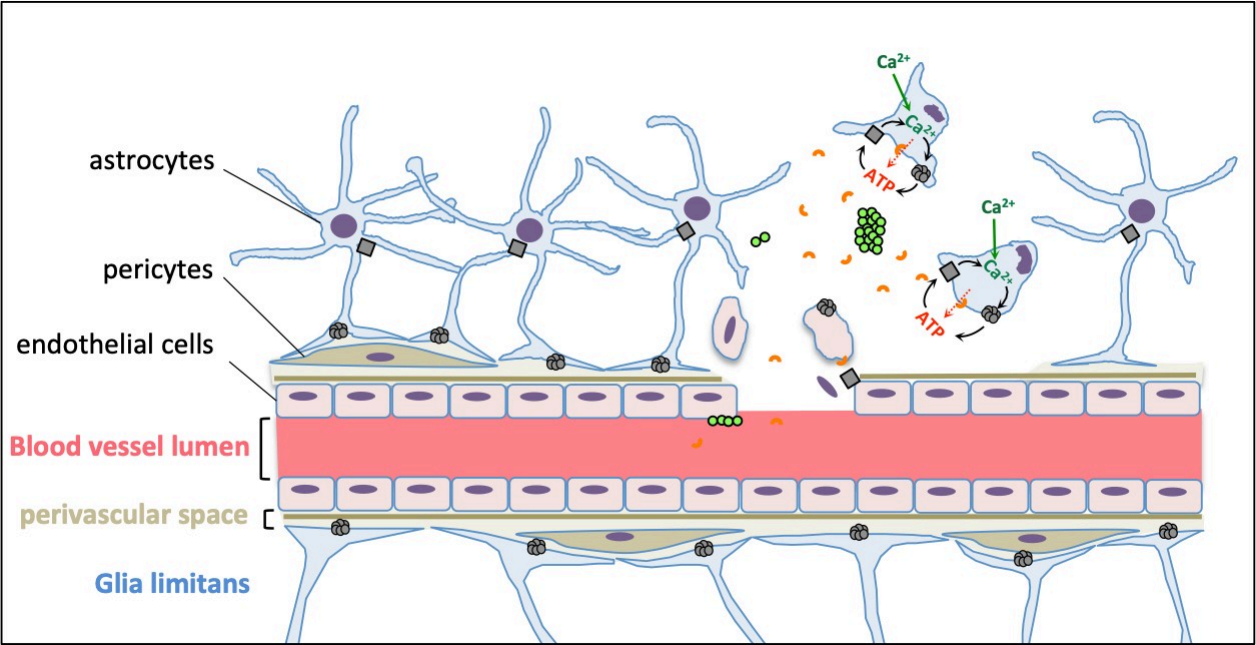
Role of aCx43 in astrocyte targeting by Ply and PN meningitis. Astrocytes regulate the BBB function through their end-feet contacting blood vessels. The BBB is represented with major cell types of the vascular unit and the different endothelial, perivascular space and glia limitans compartments. Crossing of the BBB by PN is associated with the destabilization of blood vessels seals. Secreted Ply induces the release of ATP (red arrow) in the extracellular space by forming small pores or “arcs” in the plasma membrane of astrocytes (orange half circles). Black arrows: ATP stimulates Ca^2+^ signaling via purinergic receptors (grey box). Increase cytosolic Ca^2+^ activates the opening of Cx43 hemichannels (grey circles) further amplifying ATP release and cytosolic Ca^2+^ increase, resulting in the destruction of astrocytic processes, plasma membrane permeabilization and eventually astrocytic death linked to Ca^2+^ influx (green arrow) and overload. Disruption of astrocyte-endothelial cells regulation destabilizes the BBB, favoring PN translocation and growth in the brain cortex due to local perfusion of blood vessel luminal content.

Based on our findings, we propose that during the early stages of PN meningitis, PN triggers loss of vascular endothelial cell integrity (Fig 7). The PN-secreted Ply further favors this loss of integrity by targeting astrocytes in a process dependent on aCx43 hemichannels and eATP release (Fig 7). Leakage from the brain vasculature may also provide with blood nutriments favoring PN intracortical growth in aCx43-proficient but not in deficient mice. Future works is required to evaluate the role of secreted PN products and hemichannel-mediated paracrine signaling at the various stages of bacterial meningitis.

## Materials and methods

### Ethics statement

Experiments and techniques reported here complied with the ethical rules of the French agency for animal experimentation and with the Institute of Drugs, Toxicology, Chemistry, and the Environment animal ethics committee (Paris Descartes University, Agreement 86–23).

### Bacterial strains, cell lines, and reagents

The *ply* isogenic mutant from the *S*. *pneumoniae* serotype 4 clinical isolate TIGR4 was a kind gift from Andrew Camilli (Tufts University, Boston, USA). Bacteria were grown in Todd Hewitt Broth containing (#BD249240, Thermofisher) 0.5% Yeast Extract at 37°C (#210929, Thermofisher) and plated on Columbia blood agar plates (# 43041, Biomérieux, France). Primary astrocytes were derived from mice forebrains as previously described [63]. HeLa cells (ATCC CCL-2) were from ATCC, and the stable HeLa cell line expressing human Cx43 were described previously [46]. Cells were grown in DMEM (Dulbecco's Modified Eagle Medium, #10567–014, Thermofisher) containing 10% fetal calf serum in a 37°C incubator supplemented with 10% CO_2_. The anesthetics Imagen (Ketamin) was from Merial and Rompun (Xylazin) was from Bayer Heathcare. The rabbit polyclonal anti-pneumococcal serotype 4 capsular antibody was from Statens Serum Institute, Copenhagen, Denmark). The mouse monoclonal anti-GFAP (Glial Fibrillary Acidic Protein) antibody (# G3893) and Alexa Fluor 563-conjugated isolectin IB4 (# I21412), secondary goat anti-mouse IgG antibody conjugated to Alexa Fluor 488 (#A11029), goat anti-rabbit IgG conjugated to Alexa Fluor 555 (A21424), and Alexa Fluor 633 Phalloidin (#A22284), Fluo4-AM calcium indicator (#F14201), calcein-AM (#C3100MP) were from Thermofisher Scientific. 2-(4-Amidinophenyl)-6-indolecarbamidine dihydrochloride, 4’, 6-Diamidino-2-phenylindole dihydrochloride (DAPI, #D9542), hexokinase (#9001-51-8), carbenoxolone (# C4790) were from Sigma Aldrich.

### Cloning and purification of recombinant Ply

Ply was cloned into the TOPO-TA cloning vector pET101D (#K10101, Thermofisher) using the following primers: 5’-CACCATGGCAAATAAAGCAGTAAATGAC-3’ and 5’-GTCATTTTCTACCTTATCCTCTACCTGAGG-3’. The insert was verified by DNA sequencing. Purification of recombinant Ply was performed using Talon resin (#PT1320-1, Clontech Laboratories Inc.) affinity chromatography from freshly transformed BL21/DE3 *E*. *coli* following the manufacturer’s instructions. Samples were store in 25 mM HEPES, 50 mM NaCl, 0.1% beta-mercaptoethanol in aliquots at -80°C and defrosted freshly before use.

### Mouse meningitis model

aCx43^-/-^ mice deficient for astrogial Cx43 and respective proficient aCx43^FL/FL^ mice were described previously [63]. PN cultures were freshly grown to OD_600nm_ = 0.2 and resuspended in PBS buffer at a final concentration of 5 x10^8^ cfu/ml. Following anesthesia, 6–9 weeks old C57BL/6 mice were infected through intravenous retro-orbital injection by 50 μls of the bacterial suspension. At various time points post-infection, mice were anesthetized and blood was sampled for CFU determination. For the macroscopic analysis of the BBB integrity, mice were injected via retro-orbital vein with 60 μl of PBS containing 2% Evans Blue. Mice were subjected to intracardiac perfusion with 20 mls of sterile PBS using a peristaltic pump at a flow rate of 2.5 mls / mn prior to brain sampling. Sampled brains were either flash-frozen at -80°C for subsequent RNA extraction and qRT-PCR analysis, immediately homogenized for CFU determination following plating on blood agar plates, or processed for cryosection and immunofluorescence analysis.

### QRT-PCR analysis

Total RNAs were isolated from frozen brain samples homogenized in Trizol (Life Technologies) and chloroform using glass beads and the RNeasy Lipid Tissue kit (Qiagen Corp.). qRT-PCR was performed using the Superscript II reverse transcriptase kit (Invitrogen) and SYBR green PCR master kit (Applied Biosystems) and the following pairs of primers for TNF-α: 5’-GACCCTCACACTCAGATCATCTTCT-3’ and 5’-CCTCCACTTGGTGGTTTGCT-3’; IL-1b: 5’- CTGGTGTGTGCAGTTCCCATTA-3’ and 5’-CCGACAGCACGAGGCTTT-3’; I L-1Ra: 5’-CTTTACCTTCATCCGCTCTGAGA-3’ and 5’-TCTAGTGTTGTGCAGAGGAACCA-3’; vimentin:: 5’-CGGAAAGTGGAATCCTTGCA-3’ and 5’-CACATCGATCTGGACATGCTGT-3’; GFAP: 5’-GGGGCAAAAGCACCAAAGAAG-3’ and 5’-GGGACAACTTGTATTGTGAGCC-3’. The primers used for VCAM, ICAM and P-Selectin were from the QuantiTect primer assay kit (Qiagen). Results are expressed following normalization using 18S RNA.

### Immunofluorescence microscopy analysis

Sampled brains were embedded in OCT (Tissue-Tek, Torrance, CA) and frozen in isopentane at -25°C. 20 μm sagittal sections were cut from frozen brains in a cryostat and fixed in 4% paraformaldehyde for 15 min at 21°C. Serial sections were permeabilized for 60 min in PBS containing 0.25% Triton X-100 and 5% newborn goat serum, prior to incubation with primary antibodies at the following dilutions: anti-GFAP (1:500), anti-PN capsular (1:300), and AlexaFluor 568-IB4 (1:100). Alex-conjugated secondary antibodies and Phalloidin were used at a 1:200 dilution. DAPI was used at a 0.1 mg/ml final concentration. Samples were mounted in DAKO fluorescence mounting medium (DAKO Corp.). Fixed samples were analyzed using Eclipse Ti inverted microscopes (Nikon) equipped with a 60 x objective, a CSUX1-A1 spinning disk confocal head (Yokogawa) and a Coolsnap HQ2 camera (Roper Scientific Instruments), or a CSU1-W1 confocal head (Yokogawa) and an ORCA Flash4 CMOS camera (Hamamatsu) controlled by the Metamorph 7.7 software. For live calcein assays and Ca^2+^ imaging, epifluorescence microscopy was performed using a DMRIBe microscope (LEICA microsystems) using 380 nm, 470 nm, or 546 nm LED source excitation, equipped with a Cascade 512 camera (Roper Scientific) driven by the Metamorph (7.7) software. Images were analyzed using the Metamorph software.

### Cell challenge with bacterial strains and pneumolysin

Cultured cells were seeded on sterile 25 mm-diameter coverslips (Deckgläser) at a density of 2 x 10^5^ cells / well in the day before the experiments. Cells were washed 2 times with EM buffer (120mM NaCl, 7 mM KCl, 1,8 mM CaCl_2_, 0,8 mM MgCl_2_, 25 mM HEPES pH 7.3) supplemented with 5 mM glucose. Cells were incubated with freshly grown bacteria resuspended in EM buffer at a final OD_600nm_ = 0.2, or purified Ply at the indicated concentrations for 90 min at 37°C. Samples were fixed with 3.7% paraformaldehyde, permeabilized by incubation in PBS buffer containing 0.1% Triton X-100 for 4 min at 21°C and processed for immunofluorescence staining of GFAP, F-actin, and bacterial capsule.

### Calcein release assays and Ca^2+^ imaging

Calcein release assays were performed as previously described [64]. Briefly, cultured cells were seeded on sterile 25 mm-diameter coverslips (Deckgläser) at a density of 2 x 10^5^ cells / well in the day before the experiments. Cells were washed 2 times with EM buffer and loaded with calcein-AM at 3 μM final concentration in EM buffer for 30 min at 21°C. Samples were washed three times with EM buffer and placed in a observation chamber on the microscope stage at 37°C. Samples were incubated with purified Ply at 300 nM final concentration and images were acquired at 470 nm excitation every 3 minutes for 60 min. The rates of calcein release were inferred from linear fits with a Pearson correlation coefficient > 0.95.

Ca^2+^ imaging was performed as described previously [65]. Cells were loaded with the fluorescent Ca^2+^ indicator Fluo4-AM at a final concentration of 3 μM for 20 min at 21°C. Cells were washed 3 times with EM buffer and further incubated in EM buffer for 20 min prior to mounting in the observation chamber on the microscope stage. Samples were incubated with Ply at the indicated concentration. Images were acquired every 3 seconds for at least 3 min for each Ply concentration.

### Image analysis

Identical acquisition and grey levels display parameters for all samples from the same set of experiments. A minimum of 300 fields representing 4.3 x 10^4^ μm^2^ was scored for the determination of bacterial translocation in brain slices following immunofluorescent labeling for PN capsule in at least three independent experiments. The microcolony area was determined from the sum of projected confocal planes subjected to binary thresholding. For analysis of the GFAP filament network in astrocytes from brain slices, GFAP fragments were scored using the “analyze particles “plug-in in Fiji using a minimal size threshold value of 0.05 μm^2^ and a circularity index > 0.9 in area proximal to bacterial microcolonies delimited by the presence of detectable shed capsular remnants, or the rest of the sample field. Values are expressed as numbers of GFAP fragments normalized to the surface and are representative of at least 5500 fragments in10 fields from three independent experiments. The area of DAPI-stained nucleus in in vitro grown astrocytes was determined from fluorescent images acquired at a 63x objective corresponding to a single plane of the epifluorescent DMRIbe microscope, following thresholding.

### Statistical analysis

Statistical difference was analyzed with a non-parametric Wilcoxon test for CFUs determinations, qRT-PCR values, bacterial micocolony area and calcein release assays; Mann-Whitney test for the nuclear area and cell rounding; one-way ANOVA test for the assays involving cell treatment with various Ply concentrations. ​ *: p < 0.05; **: p < 0.01; ***: p < 0.001. ****: p < 0.0001.

## Supporting information

S1 Fig aCx43 does not enhance inflammation during PN meningitis in mice. 6–9 weeks old C57BL/6 mice were infected through intravenous retro-orbital with 10^7^ bacterial CFUs. **a**, at the indicated times, mice were subjected to intracardiac perfusion with buffer than Blue Evans-containing buffer prior to brain sampling. The arrows indicate Blue Evans leakage associated with macroscopic sites of BBB rupture. Scale bar = 1 mm. **b**, bacterial CFU determination in: B, brain (solid bars); cerebrospinal fluid (grey bars). N = 3, > 3 mice per determination. **c**, 6–9 weeks old C57BL/6 mice were infected through intravenous retro-orbital with 10^7^ bacterial CFUs. At 13H post-infection, qRT-PCR was performed on total RNAs extracted from brain samples using primers specific to the indicated markers (Materials and Methods). Results are expressed as average determination value in arbitrary units normalized to values obtained for 16S mRNA. CTRL: uninfected mice. + PN: mice challenged with PN. FL: mice expressing aCx43; KO: aCx43^-/-^ mice. FL: N = 6, 6 mice per determination; KO: N = 6, 6 mice per determination. Mann-Whitney. **: p < 0.01
(TIF)

S2 Fig Destruction of the brain cortical GFAP network during PN meningitis. 6–9 weeks old C57BL/6 mice were infected through intravenous retro-orbital injection with 10^7^ bacterial CFUs. Brains were sampled at 13H post-infection and 20 μm section brain slices were processed for immunofluorescence staining of the bacterial capsule (PN, green) and GFAP (gray levels). Scale bar = 5 μm. Yellow dotted and blue outlines: area associated with PN microcolony showing capsular remnants. **a**, representative projections of confocal planes. **b**, **c**, lower magnification including the field shown in “**a**” in boxed insets. **b**, BM: binary mask of GFAP staining. **c**, detection of GFAP debris outlined in red (Materials and Methods).
(TIF)

S3 Fig Astrocyte cytotoxicity and nuclear shrinkage during PN meningitis. 6–9 weeks old C57BL/6 mice were infected through intravenous retro-orbital with 10^7^ bacterial CFUs. Brains were sampled at 13H post-infection and 20 um section brain slices were processed for immunofluorescence staining. Scale bar = 5 μm. Representative projection of confocal planes. red: IB-4 endothelial staining; green: PN capsule; gray levels: DNA. Yellow dotted outline: area associated with the PN microcolony showing capsular remnants. Arrowhead: nuclear fragmentation. Arrows: nuclear shrinkage.
(TIF)

S4 Fig Role of Cx43 in PN- and Ply-mediated plasma membrane permeabilization. Parental HeLa cells or stable transfectants expressing Cx43 (HCx43) were challenged for 90 min with wild-type TIGR4 (+PN) or an isogenic *ply* mutant (+ *ply*) (**a**, **b**), or with purified Ply (+Ply) at 250 nM or the indicated concentration (**c-e**). **a**, representative phase contrast images. Cell contours are drawn in yellow in left panels. **b,** quantification of cell retraction using circularity index as a proxy. Median values are represented. > 25 cells per sample. Mann-Whitney. ****: p < 0.001. **c**, samples were fixed and processed for fluorescence staining of F-actin.
(TIF)

S1 Video 3D-Surface rendering of GFAP fragmentation during PN meningitis. 3D-reconstruction was performed following deconvolution of confocal planes using the Huygens software. Surface rendering on was performed using the Imaris software. Green: PN capsule; grey: GFAP; red: IB-4. Scale bar = 7 μm.
(MP4)
