# Supplementary figures and images for: Role of astroglial Connexin 43 in pneumolysin cytotoxicity and during pneumococcal meningitis
Source: PLoS Pathog. 2020 Dec 28;16(12):e1009152. doi: 10.1371/journal.ppat.1009152 (PMC7793270; doi:10.1371/journal.ppat.1009152)

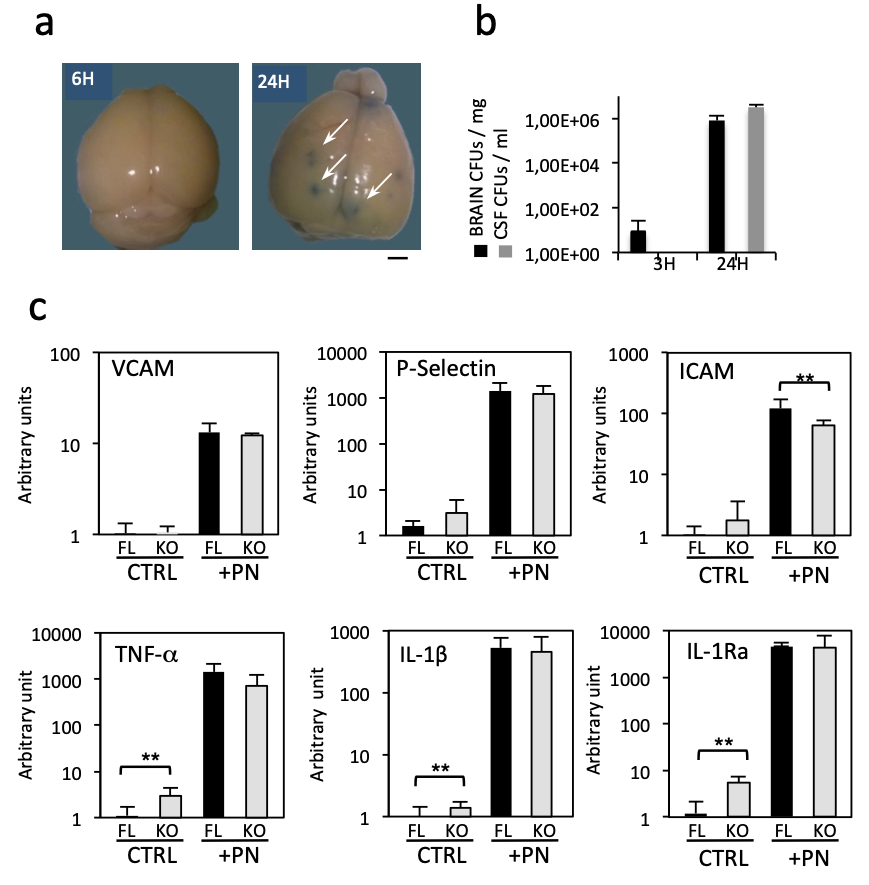

Supplement: S1 Fig — 6–9 weeks old C57BL/6 mice were infected through intravenous retro-orbital with 107 bacterial CFUs. a, at the indicated times, mice were subjected to intracardiac perfusion with buffer than Blue Evans-containing buffer prior to brain sampling. The arrows indicate Blue Evans leakage associated with macroscopic sites of BBB rupture. Scale bar = 1 mm. b, bacterial CFU determination in: B, brain (solid bars); cerebrospinal fluid (grey bars). N = 3, > 3 mice per determination. c, 6–9 weeks old C57BL/6 mice were infected through intravenous retro-orbital with 107 bacterial CFUs. At 13H post-infection, qRT-PCR was performed on total RNAs extracted from brain samples using primers specific to the indicated markers (Materials and Methods). Results are expressed as average determination value in arbitrary units normalized to values obtained for 16S mRNA. CTRL: uninfected mice. + PN: mice challenged with PN. FL: mice expressing aCx43; KO: aCx43-/- mice. FL: N = 6, 6 mice per determination; KO: N = 6, 6 mice per determination. Mann-Whitney. **: p < 0.01 (TIF) [file ppat.1009152.s001.tif]

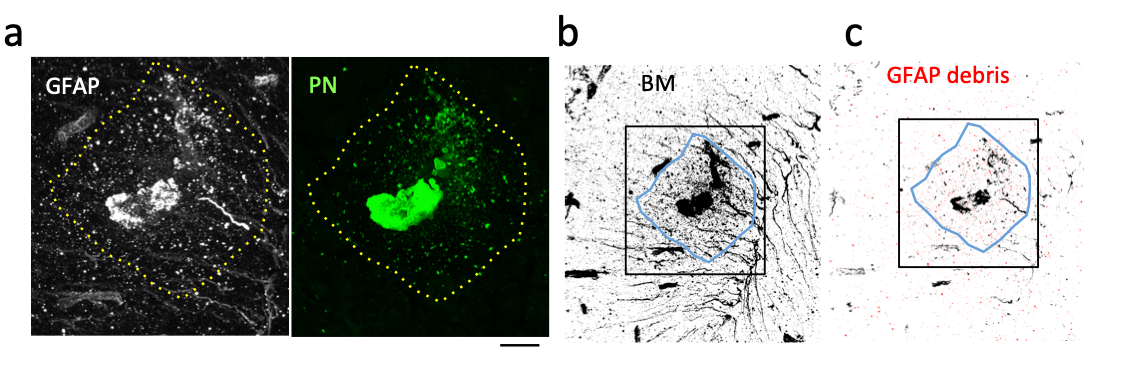

Supplement: S2 Fig — 6–9 weeks old C57BL/6 mice were infected through intravenous retro-orbital injection with 107 bacterial CFUs. Brains were sampled at 13H post-infection and 20 μm section brain slices were processed for immunofluorescence staining of the bacterial capsule (PN, green) and GFAP (gray levels). Scale bar = 5 μm. Yellow dotted and blue outlines: area associated with PN microcolony showing capsular remnants. a, representative projections of confocal planes. b, c, lower magnification including the field shown in “a” in boxed insets. b, BM: binary mask of GFAP staining. c, detection of GFAP debris outlined in red (Materials and Methods). (TIF) [file ppat.1009152.s002.tif]

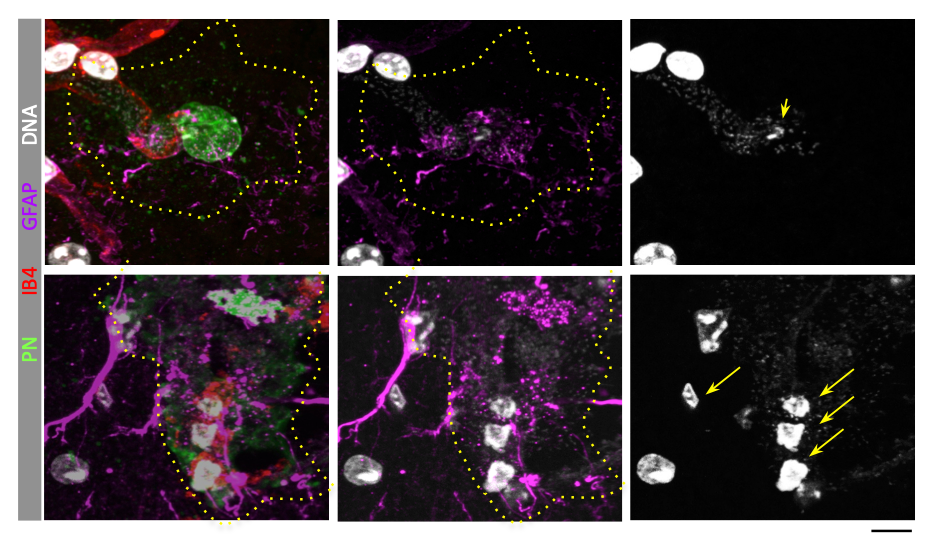

Supplement: S3 Fig — 6–9 weeks old C57BL/6 mice were infected through intravenous retro-orbital with 107 bacterial CFUs. Brains were sampled at 13H post-infection and 20 um section brain slices were processed for immunofluorescence staining. Scale bar = 5 μm. Representative projection of confocal planes. red: IB-4 endothelial staining; green: PN capsule; gray levels: DNA. Yellow dotted outline: area associated with the PN microcolony showing capsular remnants. Arrowhead: nuclear fragmentation. Arrows: nuclear shrinkage. (TIF) [file ppat.1009152.s003.tif]

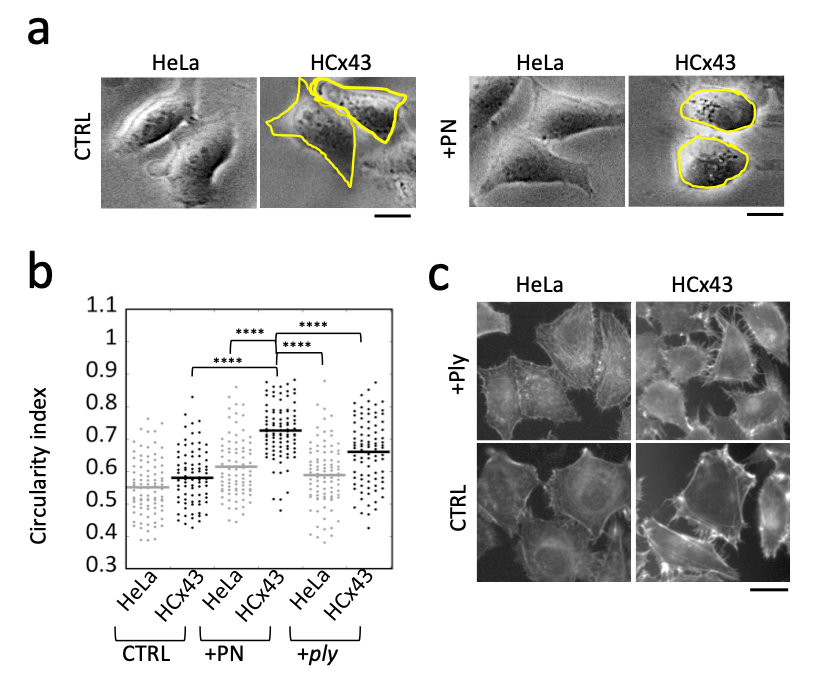

Supplement: S4 Fig — Parental HeLa cells or stable transfectants expressing Cx43 (HCx43) were challenged for 90 min with wild-type TIGR4 (+PN) or an isogenic ply mutant (+ ply) (a, b), or with purified Ply (+Ply) at 250 nM or the indicated concentration (c-e). a, representative phase contrast images. Cell contours are drawn in yellow in left panels. b, quantification of cell retraction using circularity index as a proxy. Median values are represented. > 25 cells per sample. Mann-Whitney. ****: p < 0.001. c, samples were fixed and processed for fluorescence staining of F-actin. (TIF) [file ppat.1009152.s004.tif]
